# Phyllosphere C1-microorganisms: Their interaction with plants and contribution to the global carbon cycle

**DOI:** 10.5511/plantbiotechnology.25.0122b

**Published:** 2025-09-25

**Authors:** Hiroya Yurimoto

**Affiliations:** 1 Division of Applied Life Sciences, Graduate School of Agriculture, Kyoto University, Kitashirakawa-Oiwake, Sakyo-ku, Kyoto,Kyoto 606-8502, Japan

**Keywords:** methane, methanol, *Methylobacterium*, methylotroph, phyllosphere

## Abstract

Plants emit a variety of volatile organic compounds (VOCs), including C1-compounds such as methane and methanol, which are major components of plant-derived VOCs. A group of microorganisms called methylotrophs or C1-microorganisms utilize these C1-compounds as a single source of carbon and energy and contribute to driving the global carbon cycle between two major greenhouse gases, CO_2_ and methane. C1-microorganisms inhabit the surface of the above-ground part of plants (phyllosphere), and utilize methane and methanol before they are released into the atmosphere. Among C1-microorganisms, *Methylobacterium* spp., the representative of methanol-utilizing bacteria and dominant colonizers in the phyllosphere, are known to exhibit positive effects on plants. Thus, the interactions between C1-microorganisms and plants affect not only the consumption of C1-compounds generated by plants but also CO_2_ fixation by plants. This review describes our recent understanding of the ecology and physiology of C1-microorganisms living in the phyllosphere and their application in plant biotechnology. Specifically, the ways in which these phyllosphere C1-microorganisms can be used for mitigating methane emissions as well as their application as biostimulants for increasing crop yield are discussed.

## Introduction

Interactions between plants and microorganisms can be categorized as beneficial, harmful, or neutral, and they affect not only the plant growth but also the global carbon cycle (Schenk et al. 2012; Yurimoto and Sakai 2022). Wide varieties of microorganisms inhabit various parts of plants such as the surface (as epiphytes) or the body of the plants (as endophytes) and also the soil surrounding plant roots. Due to the advent and progress in the use of analytical tools and techniques such as next-generation sequencing and omics analyses, there has been a deeper understanding of the community composition of plant-associated microorganisms and the ecology and physiology of both plants and microorganisms (Thomas et al. 2024; Trivedi et al. 2020).

The phyllosphere, the surface of above ground part of plants, including leaves, stems, flowers, acts as a habitat for a large number of microorganisms. Among these, bacteria are the most abundant, but archaea and fungi also inhabit the phyllosphere at lower numbers (Lindow and Brandl 2003). The total surface area of plant leaves on the earth has been estimated to be approximately 10^9^ km^2^, which is twice as large as the total surface area of the earth (Woodward and Lomas 2004). Assuming that the average number of bacteria in the phyllosphere is 10^7^ cells cm^−2^, the total number of bacteria in the global phyllosphere has been estimated to be 10^26^ cells (Lindow and Brandl 2003). Because some of the phyllosphere microorganisms have positive effects on plant growth and some have harmful effects such as pathogenesis, interactions between plants and phyllosphere microorganisms have a considerable impact on plant physiology. Nevertheless, the phyllosphere has long been neglected as a habitat for microorganisms and research on phyllosphere microorganisms other than plant pathogens has been less advanced compared to that on rhizosphere microorganisms.

**Figure 1:**
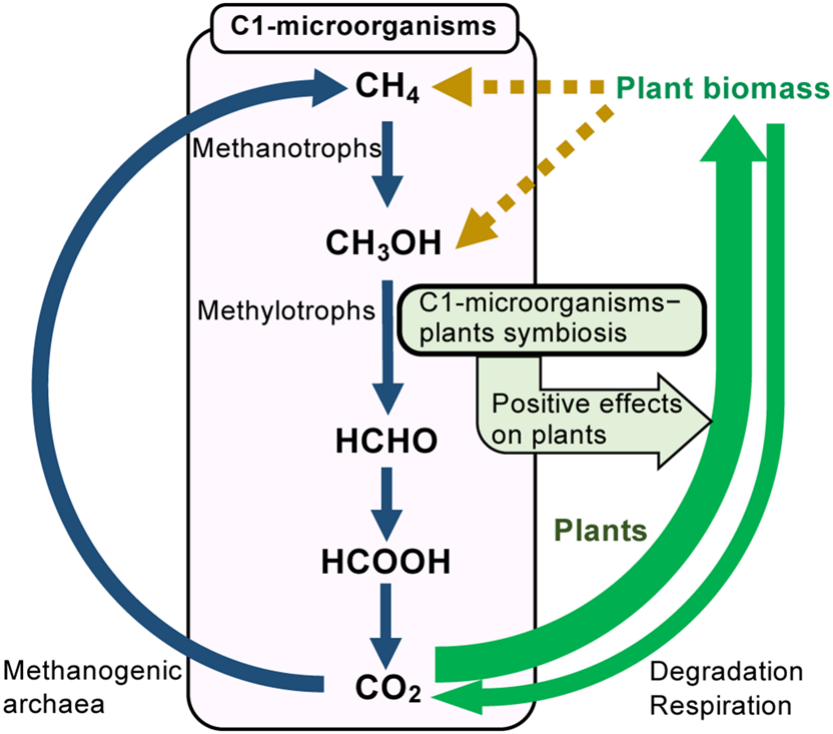
Figure 1. The global carbon cycle mediated by C1-microorganisms and plants. In the methane cycle, methane is generated from CO_2_ by methanogenic archaea, and the C1-microorganisms, including methanotrophs and methanol-utilizing methylotrophs, oxidize methane and other C1-compounds to CO_2_ (blue arrows). C1-microorganisms utilize methane and methanol produced by plants (dotted arrows). Symbiotic interactions between C1-microorganisms and plants have positive effects on plants and help increase plant biomass.

One of the dominant bacterial species in the phyllosphere is the methylotrophic *Methylobacterium* spp., called pink-pigmented facultative methylotrophs (PPFMs) (Vorholt 2012). Methylotrophs, the C1-microorganisms, are a group of microorganisms that can utilize reduced one-carbon (C1) compounds such as methane and methanol. Methane-utilizing bacteria (methanotrophs) are mostly obligate methanotrophs that use only methane (or methanol) as a carbon source (Semrau et al. 2011). On the other hand, most methanol-utilizing bacteria and yeasts are facultative methylotrophs that can also utilize compounds other than methanol, such as organic acids and sugars, as a carbon source. In the methane cycle, the global carbon cycling between the two major greenhouse gases CO_2_ and methane, C1-microorganisms play a crucial role in the biological oxidation of methane to CO_2_ (Yurimoto and Sakai 2022). Due to the ubiquitous presence of C1-compounds in nature, C1-microorganisms are widespread in various natural environments. Since it has been known that methane and methanol are released directly from plants (Keppler et al. 2006; Nemecek-Marshall et al. 1995), the phyllosphere has been recognized as a major habitat for C1-microorganisms (Iguchi et al. 2015; Vorholt 2012; Yurimoto et al. 2021b). Among C1-microorganisms, plant-associated PPFMs exert positive effects on plant health by stimulating plant growth, enhancing uptake of plant nutrients, modulating plant hormone levels, and protecting against phytopathogens (Dourado et al. 2015; Zhang et al. 2021). Thus, the interactions between C1-microorganisms and plants have great impact not only on CO_2_ fixation by plant photosynthesis, but also on emission and consumption of plant-derived C1-compounds (Figure 1).

In this review, after describing the emission of methane and methanol from plants, the ecology and the physiological functions of C1-microorganisms in the phyllosphere, including plant growth promoting traits of PPFMs, are summarized. Finally, the biotechnological application of plant-associated C1-microorganisms in reducing greenhouse gas emission and in improving crop yield are discussed.

## Emission of methane and methanol from plants

**Figure 2:**
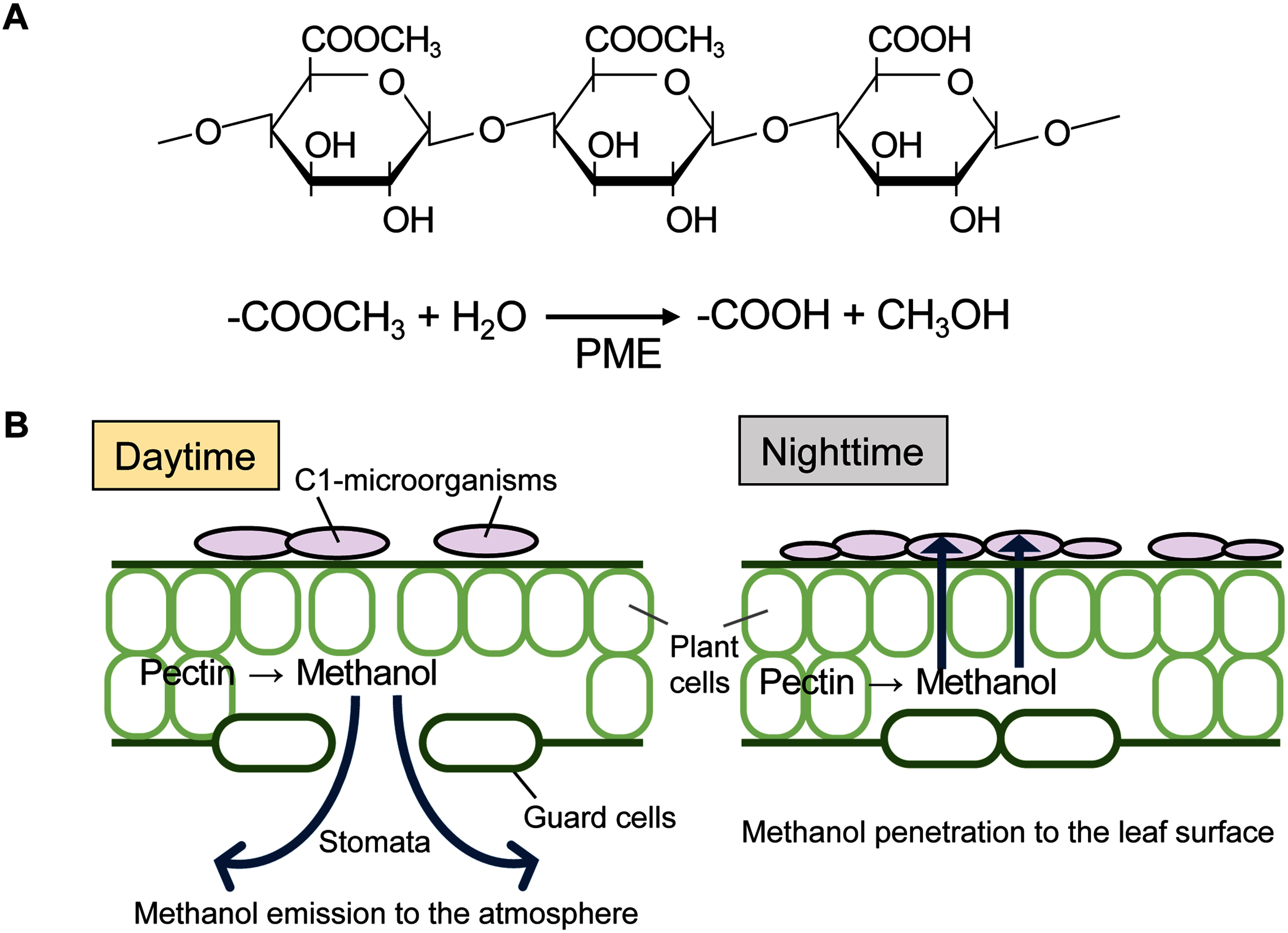
Figure 2. Methanol production from pectin and its dynamics during day and night. (A) Structure of pectin and pectin methylesterase (PME). The carboxyl groups of galacturonic acid are methylesterified and PME catalyzes the hydrolysis of methylester to generate methanol and the free carboxy group, which is used for the formation of cross-linked structures with Ca^2+^ to harden the cell wall. (B) Methanol dynamics during day and night by the opening and closing of stomata. During the daytime methanol generated from pectin is emitted to the atmosphere via stomata. During the nighttime when stomata close, methanol penetrates and concentrates on the leaf surface, which can then be utilized by phyllosphere C1-microorganisms.

Plants emit various volatile organic compounds (VOCs) to the atmosphere. Isoprene and monoterpene are the most abundant VOCs and their annual global emission is estimated to be 600–800 teragrams of carbon (Tg C) (Arneth et al. 2008). Methanol is the second most abundant VOC in the troposphere and its emission from plants is estimated to range from 75 to 280 Tg year^−1^ (Mozaffar et al. 2017). The first evidence of the direct release of methanol from leaves was reported in 1995 (Nemecek-Marshall et al. 1995). Methanol is derived from the methylester group in the cell wall component pectin (Figure 2A). Pectin is a polysaccharide whose basic structure comprises a polygalacturonic acid. Galacturonic acid undergoes methylesterification and is transported to the cell wall, where its carboxyl groups, exposed by pectin methyl esterase (PME), form cross-linked structures through interactions with Ca^2+^, thereby strengthening the cell wall (Wolf et al. 2009). Demethylesterification by PME makes the polygalacturonic acid chain more susceptible to degradation and softens the cell wall. Thus, PMEs alter the degree of pectin methylesterification to regulate the cell wall stiffness, which leads to methanol production. Methanol is also released when leaves are physically damaged by feeding or pathogen infection (Komarova et al. 2014; Midzi et al. 2022). It has been reported that methanol released from damaged leaves acts as a signal molecule to enhance pathogen resistance and induce expression of resistance-related genes in other leaves of the same plant or in leaves of neighboring plants (Dorokhov et al. 2012).

Emission of methanol from undamaged healthy leaves is affected by stomatal opening and closing, which are regulated by temperature and light. Since there is a strong correlation between methanol emission and stomatal opening, it has been assumed that methanol is emitted mainly by transpiration from the stomata during daytime (Macdonald and Fall 1993; Nemecek-Marshall et al. 1995). However, for long, the amount of methanol emission from the leaves was quantified as that released into the gas phase, and the concentration of methanol available to C1 microorganisms on the leaf surface has not been determined. Subsequently, we developed a “methanol sensor yeast cell” by using a strain that expresses the fluorescent protein Venus under the control of the methanol-inducible gene promoter of the methylotrophic yeast *Candida boidinii* and determined methanol concentration in the phyllosphere (Kawaguchi et al. 2011). The methanol sensor yeast cells were inoculated on the surface of the leaves of *Arabidopsis thaliana* and the fluorescent intensity of the cells was measured after 4-h incubation. We found that the local methanol concentration in the phyllosphere oscillated during the daily light-dark cycle; the estimated methanol concentration was higher in the dark period (25–60 mM) than in the light period (0–5 mM). These results were obtained using young *Arabidopsis* leaves 2–3 weeks after germination. In contrast to young leaves, methanol concentration on wilting or dead leaves was estimated to be greater than 250 mM and did not show diurnal oscillation. These results differ from the quantification of methanol concentration in the gas phase, which is higher during the day when the stomata are open, suggesting that C1-microorganisms utilize methanol leached into the stomatal lumen and leaf surface layer during the night when the stomata are closed (Figure 2B).

The methane emitted by plants has two sources of origin: one produced by methanogenic archaea under anaerobic conditions and released into the atmosphere through the vascular system of plants (Aulakh et al. 2000; Vroom et al. 2022), and the other produced by reactions under aerobic conditions in plant cells as reported in 2006 (Keppler et al. 2006). Methane has also been reported to act as a signaling molecule in plants, regulating gene expression related to stress responses and antioxidants (Wang et al. 2020). Methane emissions from plants, which were initially estimated to be up to several hundred Tg year^−1^, are now thought to be up to 70 Tg year^−1^ (Yurimoto and Sakai 2022). As for the mechanism of aerobic methane generation, it was reported that the methylester groups of pectin could be a precursor of methane aerobically formed by plants (Keppler et al. 2008). But recently Ernst et al. demonstrated that methane is produced from methyl radicals caused by reactive oxygen species (ROS) in the cells of all organisms, including plants (Ernst et al. 2022). Thus, verification of methane emission by plants and elucidation of the mechanism of aerobic methane formation has progressed, nevertheless, the effects on climate change and global warming have been discussed with a bias toward CO_2_, and the 6th IPCC report in 2021 does not consider methane emission from plants (Canadell and Monteiro 2021). Consequently, the effects of plant-associated C1-microorganisms on methane emission and consumption in the phyllosphere are still largely unknown.

## Distribution of methanotrophs in the phyllosphere and application in methane mitigation

Metagenomic analyses produced conflicting results on whether methanotrophs were found in the phyllosphere or not. According to some previous reports, a culture-independent metagenomic technique was able to detect methanotrophs on leaves of soybeans, rice, and *Tamarix* (Finkel et al. 2011; Ikeda et al. 2011; Knief et al. 2012), but other reports did not detect them on leaves (Delmotte et al. 2009; Yang et al. 2001). We attempted to obtain methanotrophs from various plant samples via a culture-dependent method (Iguchi et al. 2012). Using enrichment culture with methane as a single carbon source, we were able to obtain a consortium of microorganisms containing methanotrophs from various plant samples at a frequency of about 10%, indicating that methanotrophs certainly inhabit the phyllosphere. To clarify that methanotrophs can survive and proliferate on the leaf surface, a fluorescent protein-expressing strain of *Methylosinus* sp. B4S, one of the isolated methanotrophs from plant materials, was inoculated onto *A. thaliana* leaves (Iguchi et al. 2013). Fluorescent bacterial cells were observed even after 10 days after inoculation, indicating that methanotrophs can survive in the phyllosphere. It has also been recently reported that methane-utilizing bacteria live not only on the leaves of plants, but also on the bark of trees, and that the bark of trees in forests absorbs atmospheric methane, contributing significantly to global methane consumption (Gauci et al. 2024; Jeffrey et al. 2021).

In addition to terrestrial plants, both submerged and floating aquatic plants serve as a niche for methanotrophs because aqueous environments such as wetland, paddy field and lake are the major methane emission sites. This may be due to the fact that the surface of aquatic plants provides a boundary environment of methane emission and consumption. We evaluated the local methane sink in plants using plant parts sampled from various aquatic environments including the freshwater lake Biwa, Japan, to determine the potential of plants associated with methanotrophs to consume methane (Yoshida et al. 2014). The methane consumption activity of fully-submerged macrophytic algae was in the range of 3.7–37 mol h^−1^ g^−1^ dry weight, which was maximumly 370-fold higher than the terrestrial plant leaf. We also discovered that various methanotrophs inhabit duckweeds collected from Lake Biwa and that they exhibit significantly higher methane consumption activity compared to surrounding lake water (Iguchi et al. 2019). A symbiotic system reconstructed with the isolated strain *Methylomonas* sp. BLU1 and the duckweed *Spirodela polyrhiza* exhibited higher methane consumption activity than that of the strain BLU1 alone. Such a reconstructed methanotrophs-duckweeds symbiotic system would be useful for methane mitigation in rice paddies, which are the major methane emission sites.

## Ecology and physiology of PPFMs in the phyllosphere

The genus *Methylobacterium* is one of the major components of the bacterial community in the phyllosphere (Vorholt 2012). Although 11 species of *Methylobacterium* have been reclassified into a new genus *Methylorubrum* in 2018 (Green and Ardley 2018), the taxonomy of *Methylobacterium* and *Methylorubrum* has been controversial (Leducq et al. 2022). Because bacterial colonies of these genera exhibit pink color due to carotenoids, these bacteria are often called PPFMs. Since the dominance of PPFMs in the phyllosphere has been reported and their symbiotic relationship with plants has been of interest, many PPFM strains have been isolated from various plant samples. Some studies that explored their growth-promoting effects on plants and the physiological functions required for their proliferation and survival in the phyllosphere are described below.

Community composition of PPFMs in the phyllosphere is thought to be affected by plant genotype, plant age, soil type, climate and geography. Knief et al. reported that geographic factors had strong impact on the community composition in the phyllosphere (Knief et al. 2010). However, the host specificity at the species-level between PPFMs and plants has been unclear. To investigate the distribution of PPFMs on leaves, we determined the number of PPFMs (colony forming units g^−1^ fresh leaves) on various kinds of vegetable leaves planted at the same time in the same field (ca. 10 m × 10 m square) (Mizuno et al. 2012). Our results showed that not only the number of PPFMs, but also the closest relatives of the dominant isolates differed among the vegetable species, indicating that the plant species affect the population size and dominant species of PPFMs on leaves. Among the vegetables tested, including commercially available ones, the red perilla plant [*Perilla frutescens crispa* (Thunb.) Makino] yielded higher PPFM numbers (approximately 15% of the total number of bacteria on leaves) through measurements using DAPI-staining method. The PPFMs isolated from red perilla leaves planted at four geographically different sites in Japan had identical 16S rRNA sequences to that of the representative strain *Methylobacterium* sp. OR01 isolated from red perilla seeds. Furthermore, the direct transmission of *Methylobacterium* sp. OR01 labeled by an antibiotics-resistant gene from red perilla seeds to their leaves has also been observed (Mizuno et al. 2013). These results suggest that there is species-level specificity between plants and PPFMs and that this specificity is not affected by geographical factors.

Physiological functions of PPFMs related to proliferation and survival in the phyllosphere have been investigated using a representative model strain *Methylobacterium extorquens* AM1 (now reclassified as *Methylorubrum extorquens*). This strain has been used since the 1960s as a model strain for studies on methanol metabolism and production of useful compounds from methanol (Ochsner et al. 2015). *Mr. extorquens* AM1 has two types of methanol dehydrogenases (MDHs) that catalyze the first reaction of methanol metabolism: one is a Ca^2+^-dependent MDH encoded by *mxaFI* and the other is a La^3+^-dependent MDH encoded by *xoxF*. When *mxaF* or *xoxF* genes were disrupted, the mutant strains were shown to be less competitive than the wild-type strain during phyllosphere colonization, indicating that the availability of methanol is advantageous for the survival of PPFMs in the phyllosphere and that they use methanol as one of the main sources of carbon in the phyllosphere (Schmidt et al. 2010; Sy et al. 2005).

Compared to the rhizosphere, the phyllosphere is exposed to a variety of environmental factors such as temperature changes, UV radiation, drought, osmotic pressure, ROS stress, or low nutrient availability. Therefore, PPFMs must have some physiological functions to adapt to the everchanging phyllosphere environments. A general stress response regulator, PhyR, was shown to be involved in plant colonization of *Mr. extorquens* AM1 (Gourion et al. 2006, 2008). PhyR was first identified as an abundant protein produced by *Mr. extorquens* AM1 in the phyllosphere and the *phyR* mutant strain was not only impaired in plant colonization but also exhibited increased sensitivity to various stress, such as heat, UV, and ROS. Thus, the general stress response system regulated by PhyR plays an important role in adaptation to the phyllosphere environment.

Since the concentration of methanol, a major carbon source for PPFMs, fluctuates diurnally in the phyllosphere similar to light and temperature, we analyzed the function of candidates for circadian clock genes in the phyllosphere microorganisms. *Mr. extorquens* AM1 has *kaiC1* and *kaiC2* genes which are homologues of the cyanobacterial *kaiC* gene encoding a component of the KaiABC clock protein complex. While KaiC family proteins are well-conserved throughout divergent prokaryotes, studies on the physiological roles of KaiC proteins in microorganisms other than cyanobacteria have been limited. Competitive colonization tests on *A. thaliana* between the wild-type and gene-disrupted strains of *Mr. exorquens* AM1 revealed that KaiC2 and the downstream regulator LabA are necessary for the optimal colonization in the phyllosphere (Iguchi et al. 2018). Because both KaiC1 and KaiC2 are involved in temperature-dependent UV resistance as the positive and negative regulators, respectively, it is thought that KaiC1 and KaiC2 regulate gene expression to adapt to the phyllosphere environment where temperature and sunlight fluctuate diurnally.

Other physiological functions involved in phyllosphere colonization of PPFMs have been elucidated by several *Methylobacterium* strains other than *Mr. extorquens* AM1. Most PPFMs isolated from living plant materials, including *Methylobacterium* sp. OR01, have been shown to be pantothenate (vitamin B_5_) auxotrophs (Yoshida et al. 2019). *Methylobacterium* sp. OR01 is unable to synthesize β-alanine, a precursor for pantothenate synthesis, and the addition of β-alanine restores the growth on the minimal medium without panthotenate. β-alanine was detected on the *Arabidopsis* leaf surface at a concentration 100 times higher than that of pantothenate, suggesting that *Methylobacterium* sp. OR01 grows primarily by obtaining β-alanine in the phyllosphere. This suggested that PPFMs do not need to synthesize compounds that can be acquired in the phyllosphere and save energy costs required for their synthesis. Thus, most PPFMs might have become pantothenate auxotrophs during the evolution of adaptation to the phyllosphere environment.

Methylotaxis (chemotaxis to methanol) was thought to be crucial for plant colonization and vertical seed-to-leaf transmission of PPFMs, but methylotaxis of PPFMs has not been elucidated in detail. A recent study reported that methylotaxis is involved in plant colonization of PPFMs (Tani et al. 2023). *Methylobacterium aquaticum* 22A is an isolate from a hydroponic culture of a moss, *Racomitrium japonicum*, and has plant growth promoting activity (Tani et al. 2012). The results from this study showed that *Mb. aquaticum* 22A exhibited chemotaxis toward methanol, and also identified three methyl-accepting chemotaxis proteins (MCPs), MtpA, MtpB, and MtpC, responsible for methylotaxis. The triple-gene mutant of the MCPs did not exhibit methylotaxis and showed less efficient colonization on plants than the wild-type strain. These results suggested that methylotaxis contributes to efficient colonization in the phyllosphere.

## Plant growth promotion mechanisms by PPFMs

**Figure 3:**
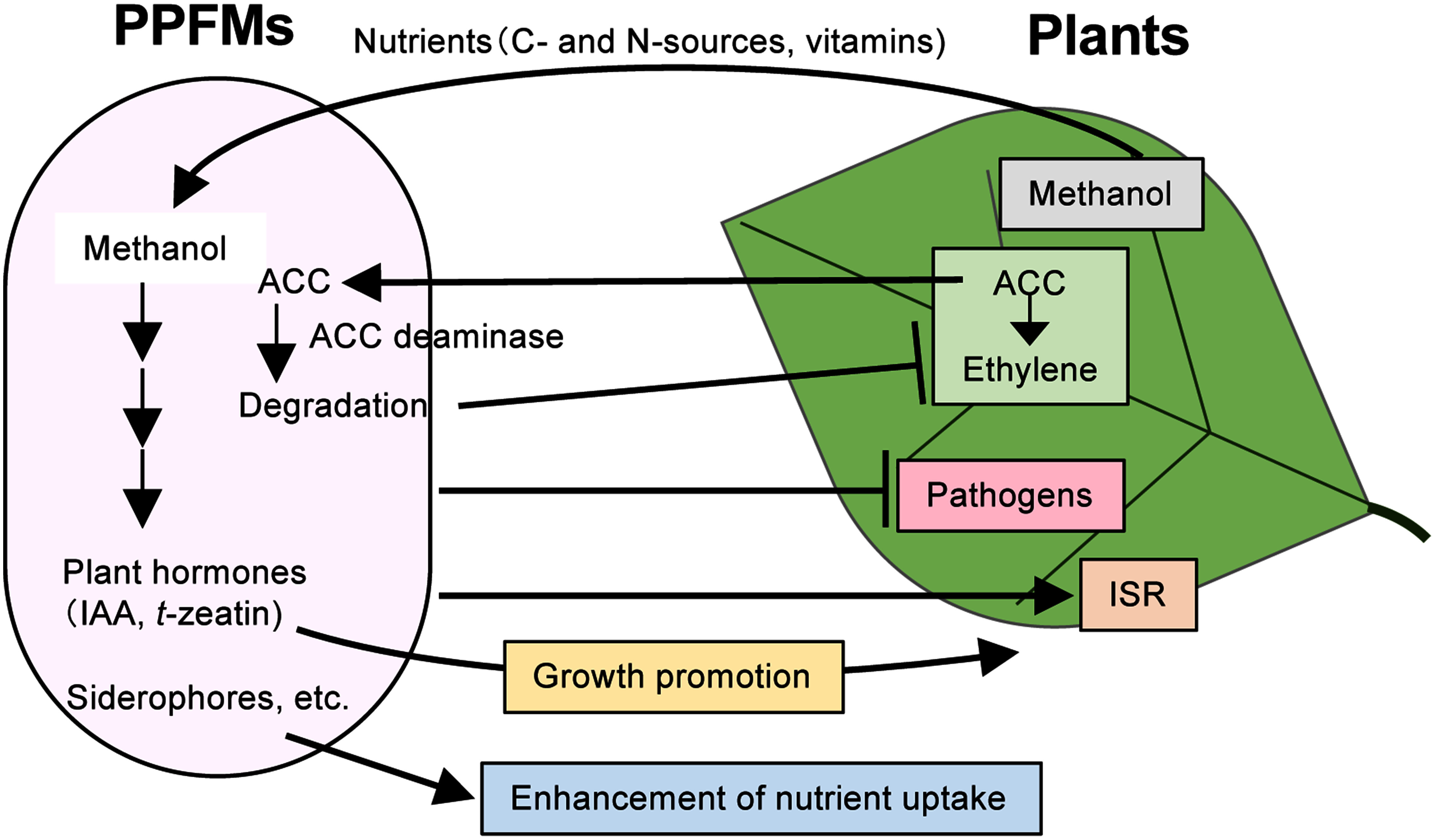
Figure 3. Symbiotic interactions between PPFMs and plants in the phyllosphere. PPFMs utilize nutrients generated by plants and produce plant hormones and other substances that have positive effects on plants. ACC: 1-aminocyclopropane-1-carboxylic acid; IAA: indole acetic acid; ISR: induced systemic resistance.

PPFMs positively affect plant health and growth, and some factors involved in plant growth promotion traits of PPFMs have been elucidated (Figure 3) (Dourado et al. 2015; Kumar et al. 2019; Zhang et al. 2021). One of the major traits for plant growth promotion of PPFMs is their ability to produce plant hormones. Many strains of PPFMs produce cytokinin (*t*-zeatin) and auxin (indole 3-acetic acid, IAA) and genome sequences of *Methylobacterium* spp. revealed the presence of genes involved in biosynthesis of these plant hormones (Koenig et al. 2002; Omer et al. 2004; Palberg et al. 2022). PPFMs can inhibit ethylene synthesis of plants by the degradation of 1-aminocyclopropane-1-carboxylic acid (ACC), a precursor of ethylene in plant cells, catalyzed by ACC deaminase (Hardoim et al. 2008). Because ethylene negatively regulates plant growth, degradation of ACC by ACC deaminase reduces the ethylene level in plants and promotes plant growth.

PPFMs enhance the nutrient uptake by plants not only in the phyllosphere but also in the rhizosphere. Several *Methylobacterim* sp. strains produce siderophores, which have high affinity for iron (Idris et al. 2004). The bacteria produce siderophores for the solubilization and uptake of iron for themselves, which also results in the enhancement of iron uptake by plants. A previous report suggested that *Mb. aquaticum* 22A produces the staphyloferrin B-like (sbn) siderophore that is involved in the uptake of not only iron, but also of lanthanide, which is required for *xoxF*-type MDH (Juma et al. 2022). *Mb. aquaticum* 22A promoted the growth of duckweed (*Lemna gibba* p8L) under iron-limited conditions, but the mutant strain that could not produce siderophore suppressed plant growth. PPFMs are involved in the solubilization of insoluble phosphates such as phytic acid (Agafonova et al. 2013). *Mb. oryzae* produce acid phosphatase, phytase, and carbon-phosphorus lyase for phosphate solubilization and contribute to phosphate uptake by plants (Kwak et al. 2014). PPFMs are also involved in nitrogen fixation. Several PPFM strains are reported to have nitrogenase activity that catalyzes the reduction of N_2_ to NH_3_ (Jourand et al. 2004; Madhaiyan et al. 2015). *Mb. nodulans* has *nifH*, which encodes nitrogenase, as well as *nodA* that is involved in nodule formation (Jourand et al. 2004). In addition, PPFMs are also known to inhibit infection by plant pathogens by producing antibiotic compounds against them, by suppressing their growth through competition for nutrients, and by enhancing plant immunity through induced systemic resistance (ISR) in plants (Ardanov et al. 2012; Dourado et al. 2015; Madhaiyan et al. 2006b; Zhang et al. 2021).

## Application of PPFMs as biostimulants to improve crop yields

A variety of microorganisms, such as rhizobia and mycorrhizal fungi, have been utilized as biostiumulants, which promote plant growth or improve crop yields (Jack et al. 2021). Most of the microbial biostimulants developed so far have been applied to the soil with viable microorganisms. Because the rhizosphere is rich in nutrient compounds for microorganisms, the microbial biostimulants have to compete with a wide variety of microbial species in the soil. Because the soil environments vary greatly from place to place and the composition of microbial community is also diverse in each environment, it is difficult for the microbial biostimulants to survive in the rhizosphere and achieve their effects on plants. On the other hand, the phyllosphere is considered to be an oligotrophic environment compared to the rhizosphere, and the population of microorganisms in the phyllosphere is smaller compared to that in the rhizosphere. Therefore, microorganisms naturally residing in the phyllosphere are believed to survive more easily and effectively exert biostimulant effects on plants. In this regard, plant growth promoting PPFMs, which utilize methanol derived from plants as a carbon source and dominantly colonize the phyllosphere, can be used as biostimulants in the phyllosphere.

There have been many reports on the effect of PPFMs that were inoculated on seeds on the plant growth, i.e., enhancement of germination rate, seedling growth and total biomass (Kumar et al. 2016). But foliar spraying of PPFMs would be more easily applicable for the practical use of PPFMs in agriculture compared to seed-inoculation of PPFMs due to the merits of phyllosphere PPFMs mentioned above. Indeed, the effectiveness of foliar spray of PPFMs was reported for the growth promotion of various plants (Chinnadurai et al. 2009; Madhaiyan et al. 2006a). However, most studies have been conducted under laboratory conditions or pot-scale cultivation conditions and evaluated the increase of total plant biomass rather than that of yields of fruits or crops. Although there has been several reports that foliar spray treatment of PPFMs increased rice grain yields in pot culture experiments (Madhaiyan et al. 2004; Nysanth et al. 2019), there has been no reports on the large-scale field test of this method at the practical level.

Recently, we reported that improvement of rice crop yields was achieved by foliar spraying of PPFMs in a commercial paddy field (Yurimoto et al. 2021a). The improvement of the crop yield of a sake-brewing rice cultivar Hakutsurunishiki was confirmed for over a 5-year period. The results showed that foliar spraying of living cells of *Methylobacterium* sp. Rst, which was originally isolated from the rice stem sample, as well as dead cells and cell-wall polysaccharides increased the rice crop yield. After optimization of the timing of foliar spraying, a one-time spray of dead cells after heading date was found to be most effective, resulting in 7% increase in the rate of ripening and 16% increase in the unit yield. This yield-increasing effect corresponded to an increase of about 5 g of polished brown rice per rice plant per application of about 0.6 mg of dead cells, indicating that foliar spraying of PPFM had excellent biostimulant functions. It is noteworthy that the PPFM seed inoculation or foliar spraying of at the early plant growing stage enhanced shoot length of seedlings and leaf greenization but the final crop yield was decreased. Although the underlying mechanism of this effect remains unclear, it is hypothesized that not the plant-growth promoting effects of living PPFM cells but the attachment of PPFM cell wall polysaccharides to rice plant leaves may stimulate photosynthesis or the plant’s immune system, thereby enhancing the nutrient translocation to rice grains. The fact that dead PPFM cells had biostimulant activity makes it easier to manufacture biostimulants and control their quality during transportation and storage. Furthermore, the dead cells can be sprayed as a mixture with pesticides, making it highly versatile for smart agriculture and their use in large-scale fields. On the other hand, PPFMs can be cultivated at high cell density using methanol as a carbon source (Riesenberg and Guthke 1999), which is a promising carbon resource produced not only from natural gas but also from biomass or CO_2_ by carbon-neutral processes (Pfeifenschneider et al. 2017; Schrader et al. 2009). Therefore, the use of PPFM biostimulants produced from methanol could contribute to low-carbon and circular economy.

## Concluding remarks

In this paper, our current understanding of the physiology and application of C1-microorganisms that colonize the phyllosphere and their contribution to global carbon cycle were described. Interactions between C1-microorganisms and plants significantly contribute to both the oxidation of methane and methanol to CO_2_ in the methane cycle and the enhancement of CO_2_ fixation by plants (Figure 1). Although this paper mainly introduced the physiological functions of C1-microorganisms in the phyllosphere, there are still many unknown mechanisms regarding the principles of symbiotic interactions between phyllosphere C1-microorganisms and plants. In particular, the effects of C1-microorganisms on the release of C1-compounds from plants into the atmosphere and their influence on the global carbon cycle and climate change need to be unraveled. Elucidation of these factors is expected to lead not only to the understanding of the mechanism of the global carbon cycle and the development of technologies to reduce greenhouse gas emissions, but also to the development of agricultural technologies to increase biomass yields by phyllosphere C1-microorganisms. The practical use of the symbiotic interactions between C1-microorganisms and plants might facilitate explorations of the use of these organisms in agriculture and in environmental technology.
